# Respiratory diseases in patients with bed bugs

**DOI:** 10.1111/crj.13447

**Published:** 2021-09-13

**Authors:** Johnathan M. Sheele

**Affiliations:** ^1^ Department of Emergency Medicine Mayo Clinic Jacksonville Florida USA

**Keywords:** albuterol, asthma, chronic obstructive pulmonary disease, *Cimex lectularius*, wheezing, bronchospasm

## Abstract

**Introduction:**

Some arthropods such as cockroaches can exacerbate asthma, but it is unknown is this is true of bed bugs.

**Objectives:**

The objective of this work is to determine if bronchospastic diseases like asthma and chronic obstructive pulmonary disease (COPD) would be higher for ED patients who have bed bug infestation compared with patients who do not have bed bug infestation.

**Methods:**

A case–control study was performed with 332 adult emergency department (ED) patients with bed bug infestation and 4952 without infestation. Univariable and multivariable regression analysis was performed.

**Results and Conclusion:**

Patients with bed bug infestation were not more likely to have a past history of or an ED diagnosis of asthma or chronic obstructive pulmonary disease (COPD). However, bed bug infested patients were significantly more likely to undergo chest radiography, be admitted to the hospital, and receive albuterol in the ED (*P* < 0.05). Infested patients receiving albuterol in the ED were more likely to be admitted to the hospital compared with uninfested patients receiving albuterol (*P* < 0.001). Patients with an ED or inpatient diagnosis of asthma or COPD and bed bugs (compared with those without bed bugs) had significantly more ED visits during the study (*P* < 0.03). Bed bug infestations may be associated with respiratory pathology, which requires further investigation.

**Abbreviations**
CHF: congestive heart failure
COPD: chronic obstructive pulmonary disease
ED: emergency department
ESI: Emergency Severity Index
OR: odds ratio
UH: University Hospitals

## 1 INTRODUCTION

*Cimex lectularius*, the common bed bug, is a hematophagous human ectoparasite.^1^ In the United States, bed bug encounters in emergency departments (EDs) increased sevenfold between 2007 and 2010.^2^ A survey in Cleveland, OH, found that about 2% of all ED patients reported having a bed bug infestation at home.^3^, ^4^ Household bed bug infestations can range from a few insects to many thousands.^5^ Bed bugs feed every few days and defecate close to their host.^5^ The feces of *C. lectularius* contain a large amount of histamine, which is one of six known bed bug aggregation pheromones.^6^, ^7^, ^8^ Houses infested with bed bugs have significantly higher histamine levels (0.546 μg/mg dust) than uninfested houses (<0.025 μg/mg dust) (*P* < 0.001), and these high levels persisted in the environment even after the house was heat treated.^9^

Histamine is a central mediator of asthma and allergic reactions. The cross‐linking of immunoglobulin E on mast cells causes degranulation and histamine release, and histamine is a central mediator of asthma and allergic reactions. Histamine causes bronchoconstriction, increased secretion of mucus, increased endothelial permeability, rhinorrhea, sinus congestion, and sneezing.^10^ Histamine nebulized at 0.39 to 1.28 μg/min (0.3–8.0 mg/ml administered at 0.13–0.16 ml/min) can be used clinically to compare bronchial hyperreactivity between patients who have asthma and patients who do not.^11^

Asthma is one of the most common chronic lung diseases in children. Allergens trigger asthma attacks in 60% to 90% of children and in 50% of adults, and about 80% of patients with asthma have positive results on allergen skin tests.^12^ Exposure to indoor allergens, such as cockroaches, dust mites, pollen, pet dander, and mold, can lead to atopy and asthma, and exposure to cockroaches and dust mites increases asthma morbidity.^12^, ^13^ Sensitization to cockroaches is the strongest risk factor for the development of asthma in low‐income urban populations worldwide,^13^, ^14^ and cockroaches and dust mites are among the most important causes of asthma exacerbations in urban, low‐income, and minority communities.^15^, ^16^

The prevalence of cockroach allergy ranges between 17% and 41% in the United States, and about 85% of urban homes have detectable levels of cockroach allergens.^12^, ^14^ An estimated 70% of urban children and 35% of all persons with asthma are sensitized to cockroaches.^12^, ^14^ Children sensitized to cockroaches had 78% more hospital visits, more wheezing, and more school absenteeism compared with children with asthma who had negative skin test results to cockroach allergens.^12^, ^17^ In homes of children with asthma, when the number of cockroaches is aggressively decreased, asthma health outcomes greatly improve.^18^ No studies have directly evaluated whether bed bugs similarly cause asthma or exacerbate it.

With high numbers of bed bug infestations in some low‐income and urban communities, it is important to know whether bed bugs—by their feeding, detritus, histamine production, or a combination of factors—are associated with respiratory illness similar to what has been observed with cockroaches. The objective of this study was to determine if respiratory diagnoses, particularly bronchospastic diseases like asthma and chronic obstructive pulmonary disease (COPD), along with the use of albuterol would be higher for ED patients who have bed bug infestation compared with patients who do not have bed bug infestation.

## 2 MATERIALS AND METHODS

There is no *International Classification of Diseases* code for bed bugs. University Hospitals (UH) information technology (IT) created a bed bug data set by identifying patients ≥18 years of age seen in one of nine UH EDs between February 1, 2011, through February 1, 2017, that had the words “bedbug,” “bed bug,” “Cimex,” or “lectularius” written in their clinical notes during that clinical encounter. These medical encounter records were examined by a study investigator to identify those encounters where a bed bug was suspected by clinical staff based on reports of a home infestation by the patient or an insect was found on the patient in the ED. Only the first ED encounter where a bed bug was noted was used for patients that had multiple subsequent encounters where bed bugs were noted. A total of 332 bed bug‐infested patients were identified, and they were matched 1:5 to 4980 ED patients that had no documented bed bug infestation. Controls were matched on age (within 1 year at the ED visit), sex, and the ED where the encounter occurred. There were 28 patients in the control group that expired in the ED and were not included in the analysis. Only the first documented set of triage vital signs and laboratory tests from the ED were included in the analysis. Incomplete or missing data were not included in the analysis. All medications and diagnoses were provided in text format which was explored using Excel spreadsheet software (Microsoft Corporation). The study was approved by the UH institutional review board (IRB). Manuscripts have previously been published from this data set.^19^, ^20^, ^21^

### 2.1 Statistical analysis

Continuous variables were summarized as mean (SD), and they were analyzed with the independent *t* test. Categorical variables were summarized as frequency (percentage) and analyzed with the Fisher exact test for (2 × 2) outcomes; otherwise, the Pearson *χ* ^2^ test was used. Stepwise regression analysis assisted in identifying the clinical variables for which there was not less than 20% missing data for use in the multivariable regression. Unless otherwise stated, all multivariable regression models accounted for age; race (Black vs. other); current or past history of tobacco use (yes vs. no/unknown); location prior to ED arrival (home; nursing or rehabilitation facility; or physician's office, clinic, surgery center, or another acute care inpatient facility); Emergency Severity Index (ESI) score (from 1 [*most urgent*] to 5 [*least urgent*]); ED disposition (admitted vs. discharged); method of ED arrival (emergency medical services, helicopter, police, private vehicle, public transportation, walking, or other); and use of chest radiography (yes vs. no). All tests were twosided, and *P* values of 0.05 or less were considered statistically significant. Statistical analysis was performed with JMP Pro 14 (SAS Institute Inc).

## 3 RESULTS

### 3.1 Patient characteristics

ED patient characteristics are summarized in Tables 1 and 2. Patients with bed bug infestation were more likely to be Black; to be unmarried; to come to the ED from home; to be admitted to the hospital; to arrive at the ED by emergency medical services, helicopter, or police; have past or current tobacco use, and have higher eosinophil counts. On univariable analysis, patients with infestation were more likely to not have a primary care physician (*P* < 0.001), to require an ED ventilator (*P* < 0.001), and to require an ED or inpatient ventilator (*P* < 0.001), but these variables were no longer significant on regression analysis (Table 2). On univariable analysis, patients with infestation (compared with patients without) had higher mean heart rates (89.7 vs. 84.4 beats/min; *P* < 0.001) and higher respiratory rates (18.8 vs. 18.2 breaths/min; *P* = 0.02). On regression analysis, patients with bed bug infestation had higher heart rates and higher ESI scores (*P* ≤ 0.03 for both).

**TABLE 1:** Demographic and ED visit characteristics of patients with or without bed bug infestation

Characteristic	Patients with infestation, no. (%) (*n* = 332)	Patients without infestation, no. (%) (*n* = 4952)	*P* value
Sex			0.95
Female	189 (56.9)	2828 (57.1)	
Male	143 (43.1)	2124 (42.9)	
Age, years			>0.99
≤40	47 (14.2)	703 (14.2)	
41–68	171 (51.5)	2548 (51.5)	
≥69	114 (34.3)	1701 (34.3)	
Race	(*n* = 331)	(*n* = 4917)	<0.001
Black	274 (82.8)	2684 (54.6)	
Other	57 (17.2)	2233 (45.4)	
Marital status	(*n* = 329)	(*n* = 4909)	<0.001
Married or life partner	47 (14.2)	1659 (33.5)	
Separated, widowed, or divorced	92 (27.7)	1260 (25.4)	
Single	190 (57.2)	1990 (40.2)	
Health insurance			0.02
Private	177 (53.3)	2949 (59.6)	
Medicare	98 (29.5)	1322 (26.7)	
Medicaid	26 (7.8)	229 (4.6)	
Unknown or no insurance	31 (9.3)	452 (9.1)	
Location before ED visit	(*n* = 330)	(*n* = 4826)	<0.001
Home	323 (97.8)	4442 (92.0)	
Nursing or rehabilitation facility	5 (1.5)	140 (2.9)	
Physician's office, clinic, surgery center, or inpatient at another acute care facility	2 (0.6)	244 (5.1)	
Transportation to ED	(*n* = 270)	(*n* = 4920)	<0.001
EMS, helicopter, or police	166 (61.5)	1655 (33.6)	
Public transportation, walking, or other	6 (2.2)	229 (4.7)	
Private vehicle	98 (36.3)	3036 (61.7)	
Discharged from ED	68 (20.5)	3117 (62.9)	<0.001

Abbreviations: ED, emergency department; EMS, emergency medical services.

**TABLE 2:** Clinical data and history for patients with or without bed bug infestation

Feature	Patients with infestation (*n* = 332)^a^	Patients without infestation (*n* = 4952)^a^	Unadjusted OR (95% CI)	*P* value	Adjusted OR (95% CI)	Adjusted *P* value
Temperature, °F	97.7 (1.31) [*n* = 332]	97.8 (1.15) [*n* = 4890]	NA	0.31	0.96 (0.87–1.06)	0.38
Heart rate, beats/min	89.7 (16.41) [*n* = 331]	84.4 (17.63) [*n* = 4900]	NA	<0.001	1.01 (1.00–1.01)	0.03
Respiratory rate, breaths/min	18.8 (4.88) [*n* = 331]	18.1 (2.74) [*n* = 4888]	NA	0.02	1.00 (0.96–1.04)	0.87
Mean arterial pressure, mm Hg	101.8 (22.01) [*n* = 330]	102.4 (17.60) [*n* = 4891]	NA	0.60	1.00 (0.99–1.00)	0.22
Peripheral capillary SpO_2_, %	97.6 (2.51) [*n* = 331]	97.6 (2.63) [*n* = 4884]	NA	>0.99	1.01 (0.96–1.06)	0.65
ESI score (from 1 to 5), points	2.9 (0.81) [*n* = 323]	2.9 (0.74) [*n* = 4751]	NA	0.34	1.90 (1.53–2.35)	<0.001
BMI	29.8 (11.68) [*n* = 308]	29.4 (8.21) [*n* = 4767]	NA	0.50	0.99 (0.98–1.01)	0.20
Has a primary care physician	28.9% (*n* = 96)	39.6% (*n* = 1961)	0.62 (0.49–0.79)	<0.001	0.85 (0.63–1.15)	0.30
Current or past tobacco use	48.2% (*n* = 160)	23.8% (*n* = 1176)	2.99 (2.39–3.74)	<0.001	1.47 (1.10–1.95)	0.009
Admitted to ICU or step‐down unit	3.6% (*n* = 12)	2.3% (*n* = 114)	0.63 (0.34–1.15)	0.13	0.94 (0.46–1.91)	0.86
Past history of asthma	12.7% (*n* = 42)	10.0% (*n* = 221) [*n* = 2206]	1.30 (0.91–1.85)	0.15	0.90 (0.58–1.38)	0.61
Past history of COPD	9.3% (*n* = 31)	7.5% (*n* = 166) [*n* = 2206]	1.27 (0.85–1.89)	0.27	1.38 (0.86–2.21)	0.18
Past history of bronchitis	1.8% (*n* = 6)	1.4% (*n* = 30) [*n* = 2206]	1.33 (0.55–3.23)	0.46	1.02 (0.36–2.83)	0.97
ED ventilator order	4.2% (*n* = 14)	1.4% (*n* = 69)	3.12 (1.73–5.60)	<0.001	1.38 (0.69–2.76)	0.36
ED or inpatient ventilator order	9.9% (*n* = 33)	4.0% (*n* = 196)	2.68 (1.82–3.94)	<0.001	1.07 (0.67–1.71)	0.76
Eosinophil count, ×10^9^/L	0.19 (0.31) (*n* = 236)	0.15 (0.19) (*n* = 2430)	1.96 (1.21–3.17)	0.006	2.17 (1.20–3.90)	0.01

Abbreviations: BMI, body mass index; COPD, chronic obstructive pulmonary disease; ED, emergency department; ESI, Emergency Severity Index; ICU, intensive care unit; NA, not applicable; OR, odds ratio; SpO_2_, oxygen saturation as measured by pulse oximetry.

^a^ Values are mean (SD) [sample size] or percentage of patients (sample size).

### 3.2 Diagnoses

#### 3.2.1 Past medical diagnoses

On univariable analysis, patients with bed bug infestation were not more likely to have a documented past history of asthma, COPD, or bronchitis (Table 2).

#### 3.2.2 ED diagnoses

On univariable analysis, patients with bed bug infestation (compared with patients without) were significantly more likely to receive an ED diagnosis of congestive heart failure (CHF) or pneumonia (*P* < 0.001 for both) but not asthma or COPD (Table 3).

**TABLE 3:** ED diagnoses for patients with or without bed bug infestation

ED diagnosis	Patients with infestation (*n* = 332)^a^	Patients without infestation (*n* = 4952)^a^	Unadjusted OR (95% CI)	*P* value
Asthma	1.2% (4)	1.6% (78)	0.33 (0.10–1.12)	0.08
COPD	2.1% (7)	1.2% (59)	1.79 (0.81–3.94)	0.19
CHF	7.2% (24)	1.8% (88)	4.31 (2.70–6.86)	<0.001
Pneumonia	5.1% (17)	1.9% (93)	2.82 (1.66–4.79)	<0.001
Dyspnea	2.4% (8)	1.2% (58)	2.08 (0.99–4.40)	0.07
Bronchitis	0.6% (2)	1.6% (80)	0.37 (0.09–1.51)	0.24
Pulmonary embolism	0% (0)	0.1% (4)	NA	>0.99
Pulmonary edema	0.6% (2)	0.4% (18)	1.66 (0.38–7.19)	0.36

Abbreviations: CHF, congestive heart failure; COPD, chronic obstructive pulmonary disease; ED, emergency department; NA, not applicable; OR, odds ratio.

^a^ Values are percentage of patients (sample size).

#### 3.2.3 ED plus inpatient diagnoses

On univariable analysis, patients with bed bug infestation (compared with patients without bed bugs) were significantly more likely to receive a diagnosis in the ED, inpatient, or both of asthma, COPD, CHF, pneumonia, dyspnea, pulmonary embolism or deep vein thrombosis, or pulmonary edema (*P* ≤ 0.001 for all) (Table 4). On regression analysis, patients with bed bugs were significantly more likely to be diagnosed with COPD (*P* = 0.01), CHF (*P* = 0.003), pneumonia (*P* = 0.006), and pulmonary edema (*P* = 0.049). Patients with bed bugs were not more likely to receive a new ED or inpatient diagnosis (i.e., no documented past history of that diagnosis for that patient in the medical record) for asthma or COPD.

**TABLE 4:** ED or inpatient diagnoses for patients with or without bed bug infestation

ED or inpatient diagnosis	Patients with infestation (*n* = 332)^a^	Patients without infestation (*n* = 4952)^a^	Unadjusted OR (95% CI)	*P* value	Adjusted OR (95% CI)	Adjusted *P* value
Asthma	14.8% (49)	8.6% (424)	1.85 (1.34–2.54)	<0.001	1.09 (0.74–1.61)	0.67
COPD	13.9% (46)	5.8% (289)	2.60 (1.86–3.62)	<0.001	1.67 (1.12–2.50)	0.01
CHF	26.8% (89)	10.4% (513)	3.17 (2.44–4.11)	<0.001	1.67 (1.20–2.32)	0.003
Pneumonia	13.3% (44)	5.5% (273)	2.62 (1.86–3.68)	<0.001	1.78 (1.18–2.69)	0.006
Dyspnea	6.6% (22)	3.0% (150)	2.27 (1.43–3.61)	0.001	NA	NA
Bronchitis	4.5% (15)	2.7% (133)	1.71 (0.99–2.96)	0.06	1.16 (0.60–2.21)	0.66
Pulmonary embolism or deep vein thrombosis	9.3% (31)	3.7% (184)	2.67 (1.79–3.97)	<0.001	1.58 (1.00–2.52)	0.05
Pulmonary edema	7.8% (26)	3.2% (159)	2.56 (1.67–3.94)	<0.001	1.66 (1.00–2.75)	0.049

Abbreviations: CHF, congestive heart failure; COPD, chronic obstructive pulmonary disease; ED, emergency department; NA, not applicable; OR, odds ratio.

^a^ Values are percentage of patients (sample size).

### 3.3 ED medications

#### 3.3.1 Corticosteroids

There were no significant differences between those with and without bed bugs and for the ED administration of corticosteroids (Table 5).

**TABLE 5:** Medications administered in the ED to patients with or without bed bug infestation

Medication	Patients with infestation (*n* = 332)^a^	Patients without infestation (*n* = 4952)^a^	Unadjusted OR (95% CI)	*P* value	Adjusted OR (95% CI)	Adjusted *P* value
Albuterol	14.8% (49)	5.6% (275)	2.94 (2.12–4.08)	<0.001	1.51 (1.01–2.27)	0.047
Corticosteroid^b^	8.4% (28)	5.9% (294)	1.46 (0.97–2.19)	0.07	0.99 (0.61–1.61)	0.97

Abbreviations: ED, emergency department; OR, odds ratio.

^a^ Values are percentage of patients (sample size).

^b^ Prednisone, dexamethasone, methylprednisolone, or prednisolone.

#### 3.3.2 Albuterol

On both univariable analysis (*P* < 0.001) and regression analysis (*P* = 0.047), patients with bed bug infestation (compared with patients without) were significantly more likely to receive albuterol (Table 5). In the ED, albuterol was administered to 6.1% of all patients (324 of 5280); of those, 56.8% (184 of 324) had a past or current ED or inpatient diagnosis of asthma and/or COPD. Among ED patients with no diagnosis of anaphylaxis, no known serum potassium greater than 6.0 mEq/L, and no ED or inpatient diagnosis of asthma or COPD, albuterol use was significantly higher among patients with bed bug infestation compared with patients without (odds ratio [OR], 4.0; 95% CI, 2.67–5.93; *P* < 0.001).

### 3.4 ED radiology

Patients with bed bug infestation (compared with patients without) were significantly more likely to undergo radiography of the chest 59.6% (*n* = 198) versus 33.8% (*n* = 1674) (*P* < 0.001), respectively, on univariable analysis and OR 1.74 (1.26–2.38) (*P* = 0.009) on multivariable regression analysis. After additional regression analysis was performed to account for a past or current ED or inpatient diagnosis of dialysis and an ED or inpatient diagnosis of pneumonia, CHF, asthma, and/or COPD, patients with bed bug infestation were still more likely to undergo chest radiography in the ED (OR, 1.50; 95% CI, 1.08–2.09; *P* = 0.02).

### 3.5 Hospital admission

On regression analysis, patients with bed bug infestation compared with those without were more likely to be admitted to the hospital if they had an ED or inpatient diagnosis of asthma (OR, 12.24; 95% CI, 3.05–49.17; *P* < 0.001), COPD (OR, 7.72; 95% CI, 2.09–28.48; *P* = 0.002), pneumonia (OR, 13.84; 95% CI, 3.16–60.67; *P* < 0.001), or given albuterol in the ED (OR, 13.13; 95% CI, 3.50–49.20; *P* < 0.001).

### 3.6 ED encounters during the study period

Among patients with an ED or inpatient diagnosis of asthma or COPD, those who had a bed bug infestation (compared with those who did not) had a significantly higher number of ED visits during the study period on both univariable analysis and regression analysis (*P* ≤ 0.03 for all) (Table 6).

**TABLE 6:** Number of ED visits for patients with or without bed bug infestation who were diagnosed with asthma or COPD

ED or inpatient diagnosis	Patients with infestation^a^	Patients without infestation^a^	*P* value^b^	Adjusted OR (95% CI)	Adjusted *P* value
Asthma	18.0 (36.3); 49	5.9 (7.4); 424	0.03	1.07 (1.03–1.12)	<0.001
COPD	12.0 (11.6); 46	6.0 (8.3); 289	0.001	1.04 (1.01–1.08)	0.009

Abbreviations: COPD, chronic obstructive pulmonary disease; ED, emergency department; OR, odds ratio.

^a^ Values are median number of ED visits during study period (interquartile range); sample size.

^b^ Wilcoxon rank sum test.

## 4 DISCUSSION

There was an association between ED patients with bed bug infestations and having respiratory complaints in the ED. While patients with bed bug infestation were not more likely to receive an ED diagnosis of asthma or COPD, they were significantly more likely to be treated with albuterol, a β‐agonist used to treat wheezing and bronchospasm in the ED. Even when accounting for potential reasons for ED administration of albuterol, such as hyperkalemia, anaphylaxis, and a diagnosis of COPD or asthma, patients with bed bug infestations were significantly more likely to be given albuterol. Corticosteroids are frequently administered in the ED to patients with asthma or COPD, but not to all patients with bronchospasm, and corticosteroids were not more common among patients with bed bugs. The data suggest that bed bugs could be associated with bronchospastic disease or wheezing in patients without existing asthma and COPD but may not be a major contributing factor for acute asthma and COPD exacerbations. However, patients with bed bugs plus asthma or COPD did have an overall higher number of ED visits during the study period compared with those without bed bugs, but clinical data on these other visits were not included in the data set.

Additional investigations are needed to follow up on our findings of bed bugs being associated with pneumonia and CHF. It is possible that variables not accounted for in our regression analyses could eventually better explain these findings.

Besides cockroaches, other insects, including moths, mosquitoes, butterflies, and silkworms, are known to cause inhalational sensitization in humans.^22^, ^23^ Some feeding insects, such as mosquitoes, can precipitate asthma,^24^ but bed bugs are rarely reported to cause bronchospasm, asthma, or anaphylaxis.^19^, ^25^, ^26^, ^27^ In a study in Egypt, *C. lectularius* extract caused positive skin reactions in a higher percentage of patients with asthma (37%–50%) compared with patients without asthma (9%–18%).^28^ A study of a survey of New York City residents found no association between recent bed bug infestations and recent episodes of asthma.^29^

### 4.1 Limitations

The patients in our database who had bed bug infestation were probably more likely to have larger chronic infestations, so identification of acute manifestations associated with an early infestation would probably be less likely. It is possible that some patients were misclassified as having or not having an infestation, and an infestation may have begun with insects from emergency medical services or the ED, but those possibilities are less likely based on previous research at UH including patient surveys, analysis of the life stages of captured bed bugs, and bed bug traps in the UH ED.^3^, ^4^, ^30^

Only 48.0% of the patients (2538 of 5284) had documented past medical histories in the data set; of those, only documented past medical histories from UH were available. Patients in the data set may have sought medical care outside of the UH network, and therefore their past medical diagnoses may not have been included in the analysis. Patients admitted to the hospital may have received more diagnoses than patients discharged from the ED. The study did not include physical examination findings, so the association between bed bugs and wheezing could not be adequately ascertained although a previous survey of UH ED patients with bed bugs found that 18% had wheezing, which is a rate higher than for undifferentiated ED patients.^30^ Not all socioeconomic variables were able to be accounted for in our retrospective chart review, and this could have affected our results.

### 4.2 Conclusions

Patients with bed bug infestation in the ED, compared with those without bed bugs, were significantly more likely to be treated with albuterol (14.8% vs. 5.6%, respectively) but not to receive a diagnosis of COPD or asthma. However, among patients with an ED or inpatient diagnosis of asthma or COPD, and who also had bed bugs (compared with those without bed bugs) were significantly more likely to be admitted to the hospital and to have a higher total number of ED encounters during the study period. Bed bug infested patients were significantly more likely to receive a chest radiograph during their ED encounter and have higher eosinophil counts than uninfested patients, but there were no significant differences in the groups regarding their initial triage peripheral capillary oxygen saturations. Some respiratory diagnoses made in the ED, including CHF and pneumonia, were also more common among patients with bed bug infestation, and additional studies are needed to further assess these associations.

## ETHICS STATEMENT

This study received institutional review board approval.

## AUTHOR CONTRIBUTIONS

The sole author designed the study, collected and analyzed the data, and wrote and critically revised the manuscript.

## FUNDING INFORMATION

The study received no external funding.

## CONFLICT OF INTEREST

The author declares that the author has no conflicts of interest.

## Data Availability

The data that support the findings of this study are available on request from the corresponding author. The data are not publicly available due to privacy or ethical restrictions.
